# Supplementary material for: Immune Responses to SARS-CoV-2 Infection and Vaccination in Dialysis Patients and Kidney Transplant Recipients
Source: Microorganisms. 2021 Dec 21;10(1):4. doi: 10.3390/microorganisms10010004 (PMC8779774; doi:10.3390/microorganisms10010004)
Supplement: Supplementary file 1 [file microorganisms-10-00004-s001.zip › microorganisms-1499348-supplementary.pdf]

Supplementary Material

Immune Responses to SARS-CoV-2 Infection and Vaccination in Dialysis Patients and Kidney Transplant Recipients

Patrick Affeldt [1#], Felix C. Koehler [1,2#], Karl August Brensing [3], Vivien Adam [1], Julia Burian [4], Linus Butt [1,2], Martin Gies [5], Franziska Grundmann [1], Steffen Hinrichs [1], Wibke Johannis [6], Nils Kalisch [1], Matthias Meyer-Delpho [7], Simon Oehm [1], Eva Platen [8], Claudia Schöler [5], Eva Heger [9], Gertrud Steger [9], Dirk Stippel [10], Aileen Ziegelhoefer [9], Thomas Benzing [1,2], Florian Klein [9], Christine Kurschat [1,2], Roman-Ulrich Müller [1,2*], Veronica Di Cristanziano[9,*]

[1] Department II of Internal Medicine and Center for Molecular Medicine Cologne, University of Cologne, Faculty of Medicine and University Hospital Cologne, 50937 Cologne, Germany

[2] CECAD, University of Cologne, Faculty of Medicine and University Hospital of Cologne, 50931 Cologne, Germany

[3] Nierenzentrum Bonn, 53175 Bonn, Germany

[4] Nieren- und Dialysezentrum Rheinbach, 53359 Rheinbach, Germany

[5] KfH-Nierenzentrum Köln-Longerich, 50737 Cologne, Germany

[6] Institute for Clinical Chemistry, Faculty of Medicine and University Hospital Cologne, University of Cologne, 50937 Cologne, Germany

[7] Dialysezentrum Siegburg, 53721 Siegburg, Germany

[8] Nierenzentrum Eifel, 53894 Mechernich, 53879 Euskirchen, 53937 Schleiden, Germany

[9] Institute of Virology, Faculty of Medicine and University Hospital Cologne, 50935 Cologne, Germany

[10] Department of General, Visceral, Cancer and Transplant Surgery, Faculty of Medicine, University Hospital Cologne, University of Cologne, 50937 Cologne, Germany.

[#] Contributed equally.

*** Corresponding authors**

Roman-Ulrich Müller, MD

Department II of Internal Medicine and Center for Molecular Medicine Cologne, University of Cologne, Faculty of Medicine and University Hospital Cologne; Cologne, 50937, Germany

Tel.: +49 221 478 30966; E-mail: roman-ulrich.mueller@uk-koeln.de

Veronica Di Cristanziano, MD

Institute of Virology, Faculty of Medicine and University Hospital Cologne, Cologne, Germany

Tel.: +49 221 478 85828; E-mail: veronica.di-cristanziano@uk-koeln.de

Baseline characteristics	BNT162b2 vaccinated dialysis patients, n=475	mRNA-1273 vaccinated dialysis patients, n=138	p-value
**Age, yrs (± SD)**	70 (± 14)	61 (± 16)	**<0.001**
**Sex, n (%)**			
Female	182 (38.3)	51 (37.0)	0.772
**Pre-existing kidney disease, n (%)**			
Glomerulonephritis	85 (17.9)	27 (19.6)	0.655
Diabetic nephropathy	68 (14.3)	35 (25.4)	**0.002**
Hypertensive nephropathy	118 (24.8)	33 (23.9)	0.824
ADPKD	31 (6.5)	11 (8.0)	0.554
Other genetic nephropathy	62 (13.1)	10 (7.2)	0.062
Unknown / other	111 (23.4)	22 (15.9)	0.062
**Haemodialysis, n (%)**	449 (94.5)	138 (100.0)	
Years of Haemodialysis (IQR)	4 (2-6)	4 (1-8)	0.387
**CAPD, n (%)**	26 (5.5)	0	**0.005**
Years of CAPD (IQR)	2 (1-4)	0	n. a.

Supplemental Table S1a: Baseline characteristics of SARS-CoV-2 naïve dialysis patients vaccinated with BNT162b2 or mRNA-1273. Abbreviations: ADPKD: Autosomal Dominant Polycystic Kidney Disease; CAPD: Continuous Ambulatory Peritoneal Dialysis; IQR: Interquartile Range; n. a.: not applicable; SARS-CoV-2: Severe Acute Respiratory Syndrome Coronavirus Type 2; SD: Standard Deviation; yrs: Years.

	Sum of squares	Df	F	p-value
**Regression**	450259224.4	3	19.770	**<0.001**
**Residual**	4198147851	553		
**Total**	4648407075	556		

**Coefficients**	**Standard Error**	**t**	**p-value**
Vaccine used	281.992	3.248	**0.001**
Assay used	220.439	-1.379	0.169
Time between sampling and vaccination	6.194	0.973	0.331

Supplemental Table S1b: Differences in SARS-COV2 IgG in SARS-COV2 naïve dialysis patients vaccinated with BNT162b2 or mRNA-1273 adjusted for used Immunoassay (RBD vs. S1) and time between sampling and vaccination by a linear multiple regression model (corrected R^2=0,092) Two vaccinations with mRNA-1273 are correlated with higher SARS-COV2 IgG compared to two vaccinations with BNT162b2

Baseline characteristics	mRNA-vaccinated dialysis patients, n=613	Convalescent dialysis patients receiving a BNT162b2 booster shot, n=38	p-value
**Age, yrs (± SD)**	68 (± 15)	69 (± 16)	0.526
**Sex, n (%)**			
Female	233 (38.0)	18 (47.4)	0.197
**Pre-existing kidney disease, n (%)**			
Glomerulonephritis	112 (18.3)	3 (7.9)	0.104
Diabetic nephropathy	103 (16.8)	8 (21.1)	0.499
Hypertensive nephropathy	151 (24.6)	10 (26.3)	0.816
ADPKD	42 (6.8)	6 (15.8)	**0.041**
Other genetic nephropathy	72 (11.7)	4 (10.5)	0.820
Unknown / other	140 (22.8)	7 (18.4)	0.527
**Haemodialysis, n (%)**	587 (95.8)	37 (97.4)	0.629
Years of Haemodialysis (IQR)	4 (2-6)	3 (2-6)	0.693
**CAPD, n (%)**	26 (4.2)	1	0.629
Years of CAPD (IQR)	2 (1-4)	13	0.444

Supplemental Table S2a: Baseline characteristics of SARS-CoV-2 naïve dialysis patients vaccinated with BNT162b2 or mRNA-1273 compared to COVID-19 convalescent dialysis patients receiving a BNT162b2 booster shot. Abbreviations: ADPKD: Autosomal Dominant Polycystic Kidney Disease; CAPD: Continuous Ambulatory Peritoneal Dialysis; IQR: Interquartile Range; SARS-CoV-2: Severe Acute Respiratory Syndrome Coronavirus Type 2; SD: Standard Deviation; yrs: Years.

	Sum of squares	Df	F	p-value
**Regression**	671958428.9	3	28.647	**<0.001**
**Residual**	4917378248	585		
**Total**	5589336677	588		

Coefficients	Standard Error	t	p-value
Booster vs. 2 mRNA vaccinations	525.822	4.749	**< 0.001**
Assay used	242.729	1.692	**0.091**
Time between sampling and vaccination	6.268	1.563	0.119

Supplemental Table S2b: Differences in SARS-COV2 IgG SARS-CoV-2 naïve dialysis patients vaccinated with BNT162b2 or mRNA-1273 compared to COVID-19 convalescent dialysis patients receiving a BNT162b2 booster shot after adjusting for used assay (RBD vs. S1) and time between sampling and vaccination in days by a linear multiple regression model (corrected R^2=0,116) *Receiving a single Booster Vaccination with BNT162b2 after infection is associated with higher SARS-COV2 IgG compared to receiving two mRNA vaccinations in dialysis patients adjusted for used Assays und time between sampling and vaccination*

Anti-RBD (BAU/ml), median (IQR), n=29	Anti-S1 (BAU/ml), median (IQR), n=29	Spearman Correlation Coefficient	p-value
390.0 (793.0)	493.5 (875.7)	0.99	<0.0001

Supplemental Table S3a: Correlation between SARS-COV2 IgG measured by S1-Antigen and RBD Immunoassay in 29 dialysis patients 30 days after vaccination with BNT162b2 (BioNTech / Pfizer). Anti-SARS-CoV-2 immunoglobulin G (IgG) targeting the spike (S) protein were detected by the semiquantitative Euroimmun anti-SARS-CoV-2 IgG ELISA on the Euroimmun Analyzer I (Euroimmun Diagnostik, Lübeck, Germany) and the quantitative IDK® anti-SARS-CoV-2 IgG ELISA (Immundiagnostik AG, Bensheim, Germany) on the DYNEX DSX® (Dynex Technologies, Chantilly, VA, USA), both using the recombinant S1 antigen of the spike protein, and the chemiluminescent microparticle immunoassays by Abbott for the quantitative detection of anti-spike RBD (receptor binding domain) IgG (SARS-CoV-2 IgG II Quant) on the automated system Alinity i (Abbott, Abbott Park, IL, United States). Quantitative IgG values were measured in BAU (binding antibody unit/ml).

N 29	Mann-Whitney-U-Test	Z	Sig. (2-tailored)
BAU/ml	392.00	-0.433	0.658

Supplemental Table S3b: Comparisons of mean SARS-COV2 IgGs measured by S1-Antigen and RBD Immunoassay in 29 dialysis patients 30 days after vaccination with BNT162b2 (BioNTech / Pfizer). Anti-SARS-CoV-2 immunoglobulin G (IgG) targeting the spike (S) protein were detected by the semiquantitative Euroimmun anti-SARS-CoV-2 IgG ELISA on the Euroimmun Analyzer I (Euroimmun Diagnostik, Lübeck, Germany) and the quantitative IDK® anti-SARS-CoV-2 IgG ELISA (Immundiagnostik AG, Bensheim, Germany) on the DYNEX DSX® (Dynex Technologies, Chantilly, VA, USA), both using the recombinant S1 antigen of the spike protein, and the chemiluminescent microparticle immunoassays by Abbott for the quantitative detection of anti-spike RBD (receptor binding domain) IgG (SARS-CoV-2 IgG II Quant) on the automated system Alinity i (Abbott, Abbott Park, IL, United States). Quantitative IgG values were measured in BAU (binding antibody unit/ml). Sig.: Significance

Spearman Correlation Coefficient	p-value
0.265	0.05

Supplemental Table S4a: Correlation of timespan between transplantation and vaccination in years and SARS-COV-2 IgG: ***Years after transplantation and SARS-COV2 IgG are barely positive correlated***

	Sum of squares	Df	F	p-value
**Regression**	1767049.688	4	4.180	**0.004**
**Residual**	8667150.479	82		
**Total**	10434200.167	86		

Coefficients	Standard Error	t	p-value
Assay used	68.481	2,089	**0.04**
MPA / MMF	79.968	-2.433	**0.017**
Age (yrs)	2.005	0.011	**0.992**
Time between sampling and vaccination	1.536	0.732	0.466

Supplemental Table S4b: Linear regression model of lacking seroconversion and immunosuppressive regimens with Mycophenolate Mofetil / Mycophenolic Acid adjusted for assay used, age and time between sampling in days and vaccination. Abbreviations: MMF: Mycophenolate Mofetil; MPA: Mycophenolic Acid; yrs: Years**.** *MPA/MMF therapy is negatively correlated with SARS-COV2 IgG after two BNT162b2 vaccination adjusted for Age, time between sampling and vaccination and assay used*

Baseline characteristics	Kidney transplant recipients, n=32
**Age, yrs (± SD)**	55 (± 13)
**Sex, n**	
Female	11
Male	22
**Immunosuppression, n**	
Tacrolimus	32
Cyclosporin A	0
Belatacept	0
mTOR	0
Azathioprine	1
MMF / MPA	27
Steroid	30
**Underlying kidney disease, n**	
Glomerulonephritis	10
Diabetic nephropathy	1
Hypertensive nephropathy	2
ADPKD	10
Other genetic nephropathy	2
Unknown / other	7
**Kidney transplantation mode, n**	
Deceased	9
Living donor	23
Living donor AB0i	5
**Vaccine used, n**	
BNT162b2 (BionTech / Pfizer)	29
mRNA-1273 (Moderna)	3

Supplemental Table S5: Baseline characteristics of SARS-CoV-2 naive kidney transplant recipients with prospectively monitored for vaccine-induced humoral and cellular immunity. Abbreviations: ADPKD: Autosomal Dominant Polycystic Kidney Disease; IQR: Interquartile Range; MMF: Mycophenolate Mofetil; MPA: Mycophenolic Acid; mTOR: mechanistic Target Of Rapamycin; SD: Standard Deviation; yrs: Years.

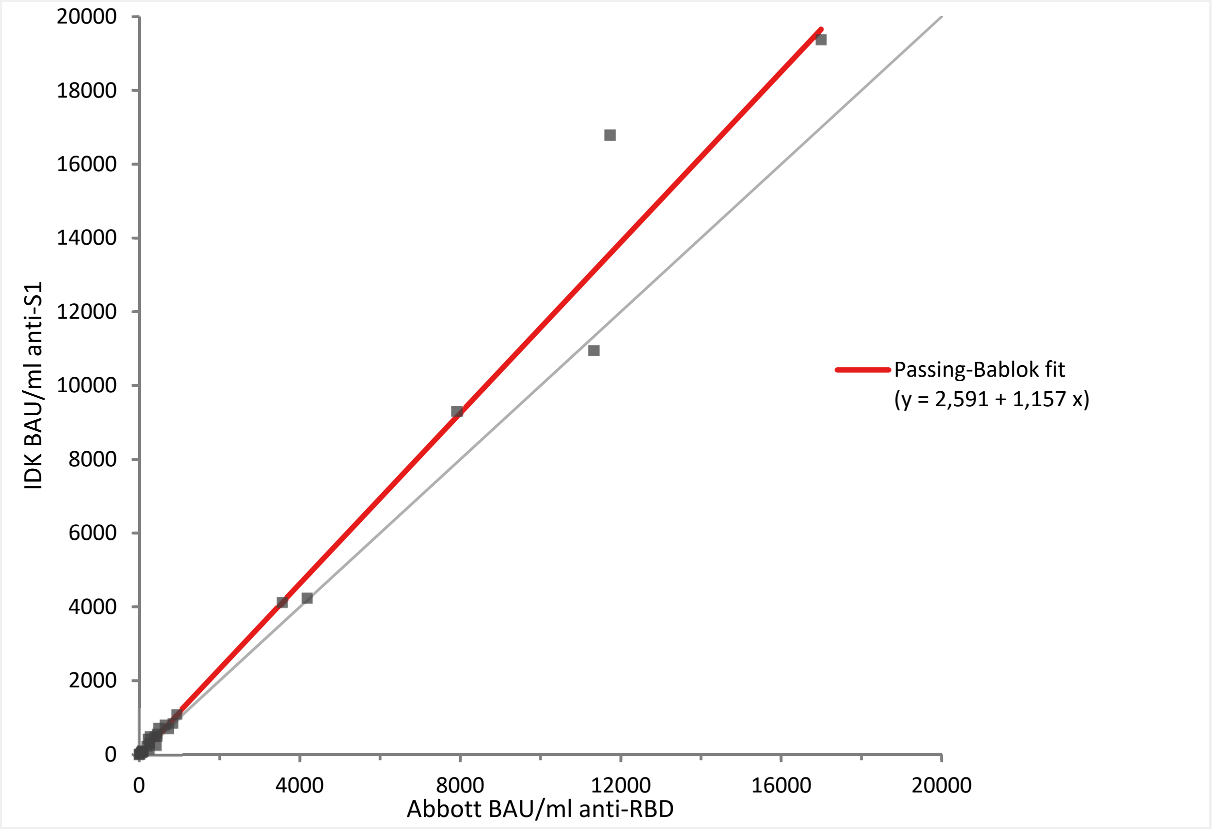

Supplemental Figure S1: Passing-Bablok-Regression for SARS-COV2 IgG Assays targeting targeting S1 or RBD: Intercept 2,6 (95%-CI: -10,7 to 14,7); Slope 1,16 (95%-CI: 1,0 to 1,3).

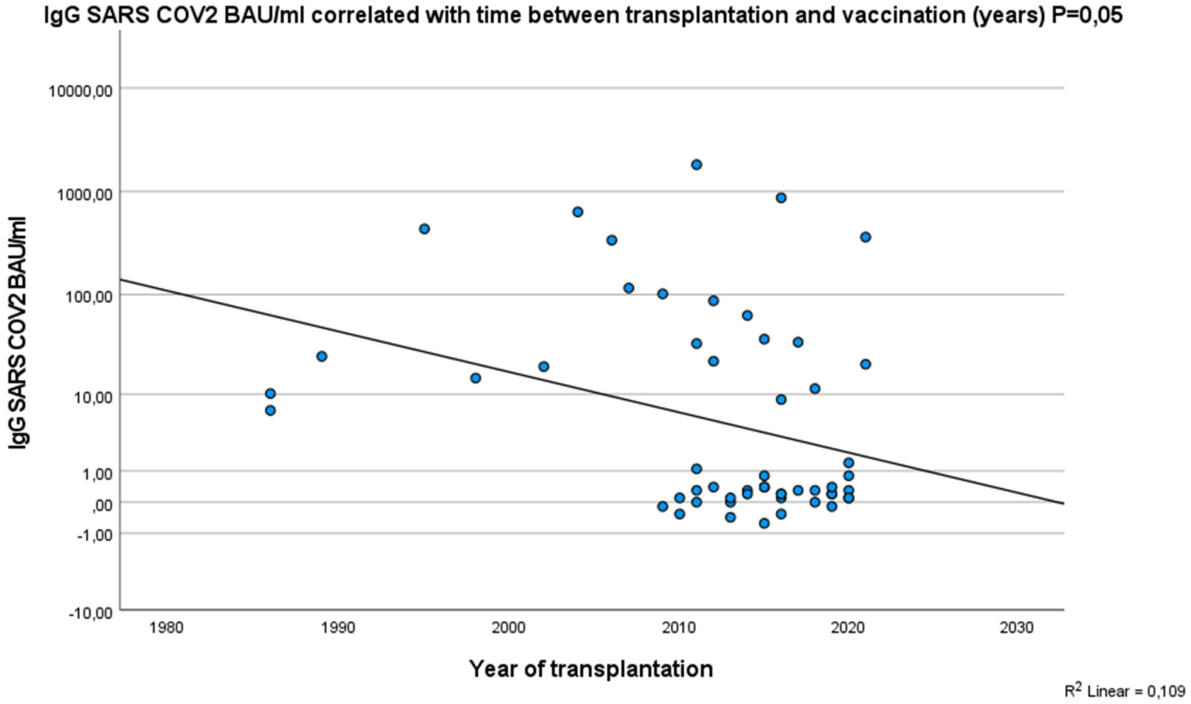

Supplemental Figure S2: IgG SARS COV2 BAU/ml correlated with time between transplantation and vaccination: X: logarithmic representation of IgG levels in BAU/ml Y: Year of Transplantation
